# Distinct tumor microenvironment landscapes in gastric cancer classified by cuproptosis-related lncRNAs

**DOI:** 10.7150/jca.79640

**Published:** 2022-12-07

**Authors:** Jianfeng Huang, Meixiang Chen, Wenguang Pei, Zhijue Xu, Jie Ning, Changyu Chen

**Affiliations:** 1 Department of General Surgery, The First Affiliated Hospital of Anhui Medical University, 218 JiXi Avenue, Hefei 230022, Anhui, China.; 2 The First School of Clinical Medicine, Southern Medical University, Guangzhou 510515, Guangdong, China.; 3 Department of Gastrointestinal Surgery, The First Affiliated Hospital of Nanchang University, Nanchang 330006, Jiangxi Province, China.; 4 Department of Vascular Surgery, Shanghai Ninth People's Hospital Affiliated to Shanghai Jiao Tong University School of Medicine, Shanghai, China.; 5 Department of Oncology, The First Affiliated Hospital of Anhui Medical University, 218 JiXi Avenue, Hefei 230022, Anhui, China.

**Keywords:** gastric cancer, cuproptosis, lncRNA, tumor microenvironment, prognosis, immunotherapy

## Abstract

Cuproptosis is a newly discovered non-apoptotic form of cell death that may be related to the development of tumors. Nonetheless, the potential role of cuproptosis-related lncRNAs in tumor microenvironment (TME) formation and patient-tailored treatment optimization of gastric cancer (GC) is still unclear. In this study, the six-lncRNA signature was constructed to quantify the molecular patterns of GC using LASSO-Cox regression model. Receiver operating characteristic (ROC) curves, C-index curves, independent prognostic analysis and principal component analysis (PCA) were conducted to verify and evaluate the model. The results showed that this risk model was accurate and reliable in predicting GC patient survival. In addition, two distinct subgroups were identified based on the risk model, which showed significant difference in biological functions of the associated genes, TME scores, characteristics of infiltrating immune cells and immunotherapy responses. We found that the high-risk subgroup was associated with immune activation and tumor-related pathways. Furthermore, compared with the low-risk subgroup, the high-risk subgroup had higher TME scores, richer immune cell infiltration and a better immunotherapy response. To accurately identify immune cold tumors and hot tumors, all samples of GC were divided into four distinct clusters by consensus clustering. Among them, Cluster 3 was identified as an immune hot tumor and was more sensitive to immunotherapy. Overall, this study demonstrates that cuproptosis-related lncRNAs could accurately predict the prognosis of patients with GC, help make a distinction between immune cold tumors and hot tumors and provide a basis for the precision medicine of GC.

## Introduction

As the fifth most common cancer globally, gastric cancer (GC) is a highly heterogeneous and aggressive digestive tract malignancy with high morbidity and mortality 1. According to the latest data (Global Cancer Statistics 2020), GC accounts for over 1 million new cases and nearly 769,000 deaths worldwide annually 2. Most GC patients are diagnosed at an advanced stage, and the 5-year survival rate is still not high even after surgery and systemic treatment 3. For patients with unresectable GC, systemic therapy, including chemoradiation, targeted therapy and immunotherapy, is their only choice 4. In recent years, immunotherapy has received great attention, and it has achieved remarkable success in the treatment of many malignant tumors. However, only one-third of patients with most types of cancer respond significantly to immune checkpoint inhibitors (ICIs) 5. Therefore, it is necessary to change the treatment strategy of GC to provide personalized treatment optimized for individual patients.

Cell death, like cell regeneration, is one of the most fundamental processes in life. Cell death can be divided into two types: programmed cell death (PCD) and necrosis. Apoptosis is a type of PCD that is controlled by genes and produced by a series of elaborate reactions 6. A recent study published in Science proposed a new form of programmed cell death: cuproptosis. Cuproptosis occurs through the direct binding of copper to lipoylated components in the tricarboxylic acid cycle. This process leads to lipoylated protein aggregation, subsequent iron-sulfur cluster protein loss and proteotoxic stress, which ultimately leads to cell death 7. Studies have shown that cuproptosis, like traditional oxidative stress-related cell death (e.g., apoptosis, ferroptosis, and necroptosis), may be closely related to the development of tumors 8, 9. In addition, in some animal models of Cu-related disorders, ionophores such as elesclomol can be used to deliver Cu and restore function, which may be related to drug resistance of cancer 10.

Long non-coding RNAs (lncRNAs) make up a large fraction of non-coding RNAs and they have several special functions, such as chromatin modification, transcription regulation, and post-transcriptional regulation 11. LncRNAs play an important role in carcinogenesis and metastasis by regulating the translation of genes or interacting with specific proteins and RNA 12, 13. In addition, recent studies have found that lncRNAs are involved in the regulation and remodeling of the tumor immune microenvironment 14, 15. LncRNAs regulate the tumor immune microenvironment in a variety of ways, help tumors escape immune surveillance, and promote tumor metastasis and drug resistance. Sun et al. identified tumor immune infiltration-associated lncRNAs that can divide patients with non-small cell lung cancer into the immune-cold group and immune-hot group and can predict the prognosis of patients and the response to immunotherapy 16. At present, the mechanism of cuproptosis in GC is still unclear, and the relationship between cuproptosis-related lncRNA expression patterns and the tumor microenvironment (TME) of GC has not been reported. Therefore, identifying cuproptosis-related lncRNAs can help us clearly understand the roles of cuproptosis and lncRNAs in the TME and immunotherapy.

In this study, cuproptosis-related lncRNAs were mined from TCGA transcript data via Pearson correlation analysis. Through univariate analysis and LASSO regression analysis, a lncRNA prognostic signature related to cuproptosis was constructed and verified for the first time. Functional enrichment, tumor microenvironment, immune cell infiltration, immune checkpoint inhibitors and drug sensitivity were analyzed to provide a robust theoretical basis for the application of immunotherapy and chemotherapy to GC patients.

## Methods

### Data acquisition and processing

The mRNA expression, lncRNA expression, somatic mutation and clinical data of 407 STAD patients were obtained from the TCGA database (https://portal.gdc.cancer.gov/repository) on 20 January 2022, which included 375 tumor samples and 32 normal samples. The expressed data were normalized to fragments per kilobase million (FPKM) 17 format for subsequent data analysis. The inclusion criteria of GC and normal samples were as follows: the survival time and clinicopathological characteristics of the patients were clear, and the gene expression and lncRNA expression of the samples were not missing. Exclusion criteria: STAD samples with overall survival time less than 30 days, unclear survival status and clinical other factors, and lack of cuproptosis-related lncRNA expression. Subsequently, the enrolled samples (337) were processed using Perl (version Strawberry-Perl-5.30.0) and R software (version 4.0.0), and they were randomly divided into the training sets and test sets through the "caret" package.

### Selection of cuproptosis-related genes and lncRNAs

A total of 16 cuproptosis-related genes were obtained by searching the latest published studies 7-10. Pearson correlation analysis was used to identify cuproptosis-related lncRNAs. Among them, | correlation coefficient| > 0.3 and p value < 0.001 were considered meaningful. Using the “limma” package 18 to screen these lncRNAs (log2-fold change (FC) > 1, p < 0.05, and false discovery rate (FDR) < 0.05), we finally obtained 634 differentially expressed cuproptosis-related lncRNAs for subsequent analysis. The differential expression of cuproptosis-related lncRNAs was expressed as a heatmap and volcano map using the “limma” and “pheatmap” packages.

### Construction and validation of the risk model

Based on the expression and clinical data in the TCGA database, univariate Cox proportional regression analysis was performed to screen the lncRNAs related to the prognosis of GC from the cuproptosis-related lncRNAs. Then, least absolute shrinkage selection operator (LASSO) regression analysis was performed to construct a predictive model for cuproptosis-related lncRNAs. The formula used to calculate each GC patient risk score was as follows: Risk Score =Ʃ (coef [lncRNA] × Exp [lncRNA]), where coef [lncRNA] represents the coefficient of each lncRNA and Exp [lncRNA] represents the expression of each lncRNA. According to the median risk score, all GC samples were separated into high-risk and low-risk subgroups. Kaplan-Meier (K-M) curves, receiver operating characteristic (ROC) curves, time-dependent ROC curves and C-index curves were used to assess the prediction performance of the risk model for the OS of GC patients in the training, test, or entire sets.

### Prognostic value of the risk model

We analyzed whether the risk model could accurately predict GC patient survival in different clinical subgroups stratified according to clinical pathological variables (including age, sex, grade, T stage, N stage, M stage). Based on the “survival” package, univariate Cox (uni-Cox) and multivariate Cox (multi-Cox) regression analyses were performed to determine whether the risk scores and clinical characteristics could be used as independent prognostic factors.

### Construction and assessment of the nomogram

A nomogram based on the risk score and independent clinical factors was constructed to predict the 1-, 3-, and 5-year OS of GC patients using the “rms” package. The calibration curve was applied to assess the accuracy of the nomogram for predicting the 1-, 3-, and 5-year OS of GC patients. Decision curve analysis (DCA) was used to appraise the clinical effect of the nomogram by calculating the net benefits of a series of risk threshold probabilities.

### Principal component analysis and gene set enrichment analysis

Based on the expression patterns of cuproptosis-related lncRNAs, the principal component analysis (PCA) was applied to reduce the dimensionality, identify the risk model, and visualize the high-dimensional data of the whole gene expression profile, cuproptosis-related genes, cuproptosis-related lncRNAs, and risk model. To investigate differences in biological functions between the high-risk and low-risk subgroups, GO and KEGG analyses were performed by gene set enrichment analyses (GSEA) software in the entire set. P < 0.01 and FDR < 0.05 were considered significant functional enrichment.

### Tumor microenvironment and immune cell infiltration

The relationship between the risk score and the TME and tumor-infiltrating immune cells (TICs) was investigated to predict their correlation. Based on the “CIBERSORT” package 19, the components of the immune and stromal cells in the TME of each GC sample were calculated to verify the differences in microenvironment features between the high-risk and low-risk subgroups. The single sample Gene Set Enrichment Analysis (ssGSEA) scores were used to evaluate the enrichment levels of 13 immune-related functions between the high-risk and low-risk groups. Additionally, the Wilcoxon signed-rank test was used to analyze the differences in immune infiltrating cell content between different groups.

### Tumor mutational burden and immunotherapy

To show the tumor mutational burden (TMB) of GC patients, the “maftools” package was used to integrate and process the somatic mutation data obtained from the TCGA database. The “limma”, “ggpubr”, “survival”, “survminer” and “maftools” packages were applied to analyze the difference in survival based on TMB between the high- and low-risk subgroups. The expression levels of common immune checkpoint molecules were compared between the high- and low-risk subgroups using the “ggpubr” package to appraise the predictive effect of the risk model for GC immunotherapy. The Tumor Immune Dysfunction and Exclusion (TIDE) score file was obtained from the TIDE website (http://tide.dfci.harvard.edu). To further predict the clinical response to ICI therapy for GC patients in different risk subgroups, TIDE analysis was performed.

### Prediction of potential chemotherapeutic drugs for GC treatment

To predict the potential chemotherapeutic drugs that may be effective in GC therapy, we calculated the half-maximal inhibitory concentration (IC50) values for each GC patient obtained from the Genomics of Drug Sensitivity in Cancer (GDSC) website (https://www.cancerrxgene.org/) 20. The “pRRophetic” 21, “limma”, “ggpub”, and “ggplot2” packages were used to evaluate their therapy response.

### Consensus clustering and precision medicine in clusters

To explore precision medicine in GC, we used the “ConsensusClusterPlus” 22 package to divide 337 GC samples into different molecular subgroups based on the expression of prognostic cuproptosis-related lncRNAs. Kaplan-Meier survival, PCA, T-distributed stochastic neighbor embedding (t-SNE), and Sankey relational diagrams were used to investigate the differences among clusters. In addition, tumor immune infiltration analysis and ICI analysis among different molecular subsets were performed.

### Statistical analysis

GC patients were divided into different subgroups according to the risk score or consistency analysis. Data between two groups were examined using a two-tailed paired Student's t-test or ANOVA. The classification variables in the training and testing tests were contrasted using the chi-square test. The correlations between subgroups, clinicopathological features, risk scores, immune infiltration levels, and immune checkpoint inhibitors were assessed using the Pearson chi-square test or Spearman rank correlation test. For all statistical analyses, R software (version 4.0.0) was utilized. P values <0.05 were considered statistically significant differences.

## Results

### Identification of cuproptosis-related lncRNAs in GC

**Figure 1:**
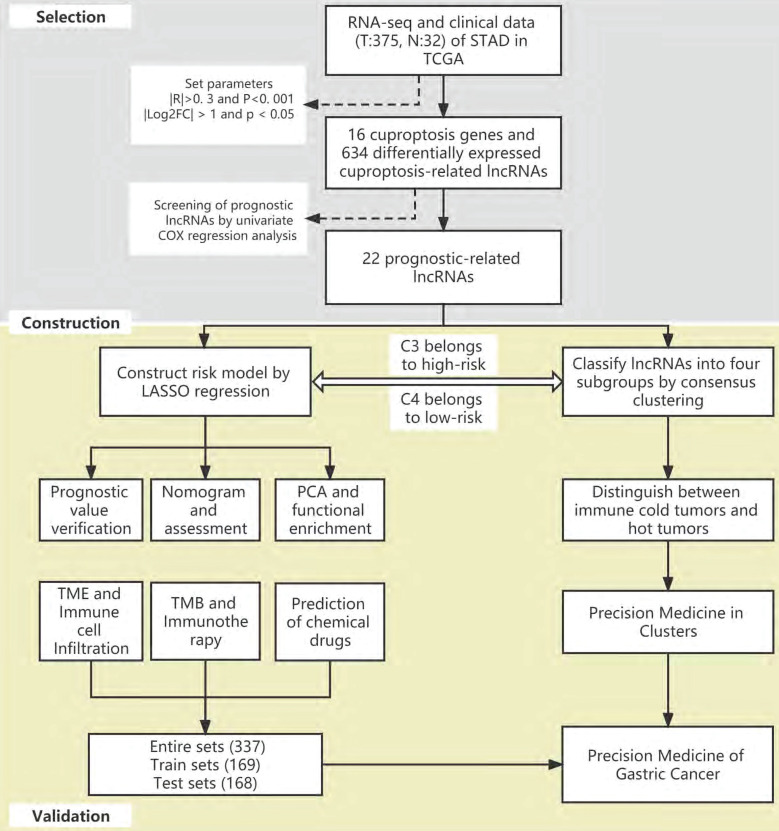
The flow of the study.

**Figure 2:**
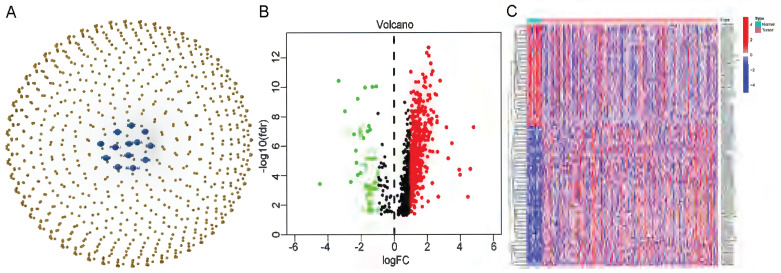
** Identification of cuproptosis-related lncRNAs in GC. (A)** The co-expression network diagram shows the lncRNAs highly related to cuproptosis genes (R>0.3, P<0.001). The volcano plot **(B)** and heatmap **(C)** show 487 differentially expressed cuproptosis-related lncRNAs.

The flow of the study is shown in Figure 1. First, we downloaded the transcriptome RNA-sequencing and matched clinical data of 407 STAD patients from the TCGA database, which contained 32 samples of normal gastric tissues and 375 samples of GC tissues. Subsequently, we annotated the transcriptome data and distinguished mRNAs and lncRNAs according to GTF files. Based on the expression of known cuproptosis-related genes and the differential expression of lncRNAs between normal and tumor samples (|Log2FC| > 1 and p < 0.05), we finally identified 634 cuproptosis-related lncRNAs by Pearson correlation analysis (|R|>0. 3 and P<0. 001) (Figure 2A). Among them, 590 lncRNAs were significantly upregulated, and 44 lncRNAs were significantly downregulated (Figure 2B, C).

### Construction and validation of the cuproptosis-related lncRNA risk model

**Figure 3:**
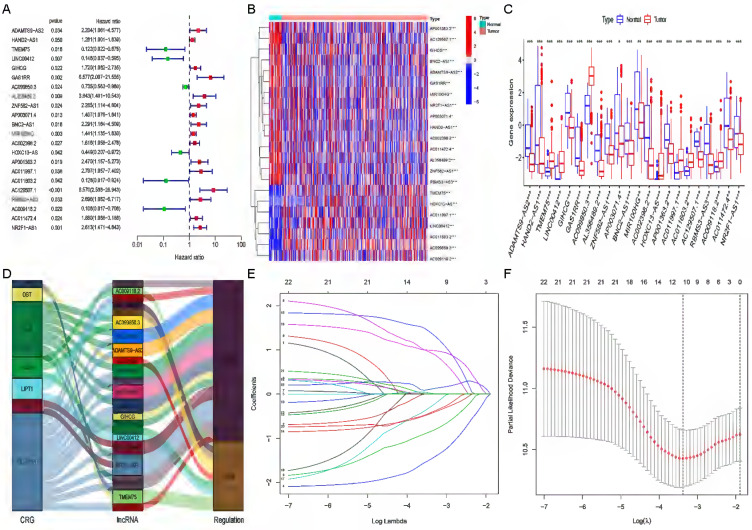
** Extraction of the cuproptosis-related lncRNA prognostic signature in GC. (A)** Forest map showing 22 prognostic lncRNAs identified by univariate Cox regression. Heatmap **(B)** and boxplot **(C)** show the differential expression of 22 lncRNAs in gastric cancer and adjacent normal tissues. *P<0.05, **P<0.01, and ***P<0.001. **(D)** The Sankey relational diagram of cuproptosis genes and cuproptosis-related lncRNAs. **(E)** LASSO coefficient profiles of cuproptosis-related lncRNAs. **(F)** Partial likelihood deviance of different numbers of variables revealed by the LASSO regression model.

We performed univariate Cox regression analysis to initially screen 22 lncRNAs closely related to the prognosis of GC patients from the 634 cuproptosis-related lncRNAs (Figure 3A). Of these, 16 and 6 lncRNAs were related to good and poor prognosis, respectively. Figure 3B and 3C show the differential expression of these 22 lncRNAs in GC and normal samples. In addition, the Sankey diagram showed the interaction of cuproptosis-related lncRNAs and cuproptosis-related genes (Figure 3D). To avoid overfitting the risk model, we performed LASSO Cox regression analysis on these lncRNAs with higher prognostic value using the “glmnet” package and selected 6 lncRNAs when the first-rank value of Log(λ) was the minimum likelihood of deviance (Figure 3E, F). A total of 337 GC samples with these lncRNA expression data and integral survival information were randomly allocated to a training set (n=169) or a test set (n=168). Based on the expression levels of the 6 lncRNAs and the corresponding weighted coefficients, the risk score of each patient in the training, test, and entire sets was calculated using the risk formula: risk score = (-1.249958 × Exp TMEM75) + (-2.274612 × Exp LINC00412) + (1.629251 × Exp GAS1RR) + (-0.820546 × Exp HOXC13-AS) + (2.079533 × Exp AC011997.1) + (1.412066 × Exp AC129507.1). The median risk score was used as the cutoff value, and GC patients in the training, test, and entire sets were divided into low-risk and high-risk subgroups.

**Figure 4:**
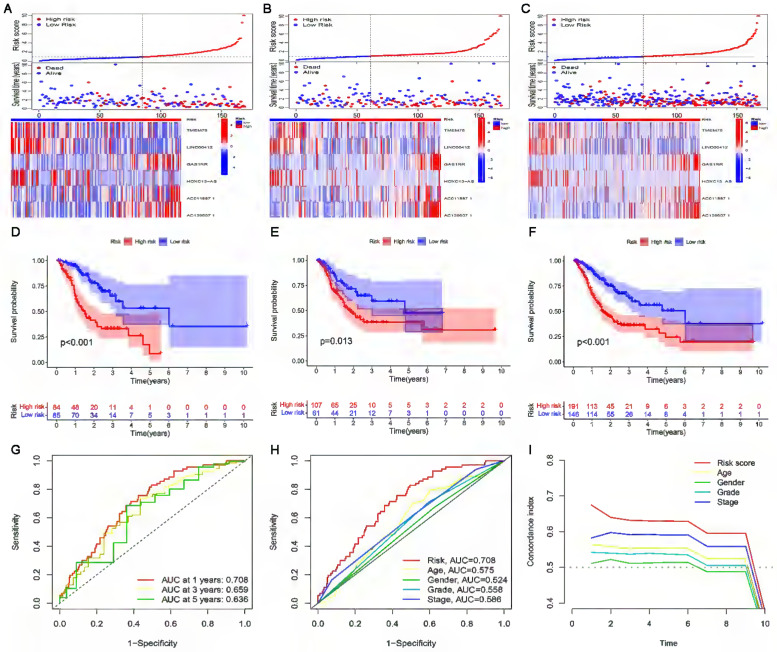
** Construction and validation of the cuproptosis-related lncRNA risk model. (A-C)** Distribution of risk score, survival status, and gene expression among patients in the training, test, and entire sets. **(D-F)** The Kaplan-Meier curves of the high- and low-risk subgroups in the training, test, and entire sets were compared.** (G)** The 1-, 3-, and 5-year ROC curves of the entire set. The 5-year ROC curves **(H)** and C-index curves **(I)** of risk score, age, sex, grade, and stage.

The risk score, survival status, and relevant lncRNA expression were compared between the high- and low-risk subgroups in the training, test, and entire sets. The results showed that the number of deaths increased significantly with increasing risk score (Figure 4A-C). The results of survival analysis also showed that the prognosis of patients in the low-risk subgroup was significantly better than that in the high-risk subgroup, regardless of the set (training, test or entire) (Figure 4D-F). In addition, the ROC curve and C-index curves were used to assess the prediction performance of the risk model for the OS of GC patients in the entire set. The values of AUC and consistency index both indicated that compared with other clinical factors (including age, gender grade and stage), the risk score was the most accurate in predicting patient survival (Figure 4G-I).

### Prognostic value of the cuproptosis-related lncRNA risk model

To further investigate the value of the risk model in clinical assessment, KM curves were used to study the relationship between risk score and survival probability in different clinical subgroups. All GC patients were stratified based on clinical pathological variables, including patients age > 65, patients age ≤65, male patients, female patients, patients with G1-2, patients with G3, patients with T1-2, patients with T3-4, patients with N0, patients with N1-3, patients with M0, patients with M1, patients with stage I-II and patients with stage III-IV. For each stratification, the OS of GC patients in the low-risk subgroup was significantly higher than that in the high-risk subgroup (Figure S1A-N). These results not only confirmed the reliability of the risk model but also indicated that the risk model was an effective predictor of survival in different subgroups of patients. In addition, univariate and multivariate Cox regression analyses were performed to determine whether risk scores could be used as independent prognostic factors. The results of independent prognostic analysis showed that the risk score was a more stable predictor (Figure S1O-P).

### Construction and assessment of the nomogram

**Figure 5:**
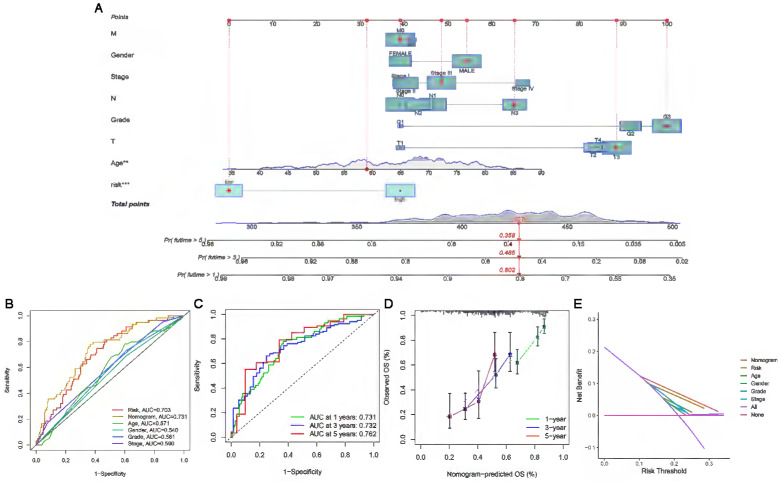
** Construction and assessment of a nomogram for predicting 1-, 3- and 5-year OS in GC patients. (A)** Construction of the nomogram comprising independent prognostic factors. **(B)** ROC curve analysis of indicated clinical factors in the entire set. **(C)** The 1-, 3-, and 5-year ROC curves of the nomogram. **(D)** The calibration curve of the nomogram for the prediction of 1-year, 3-year and 5-year survival OS probability. **(E)** Decision curve for assessment of the clinical utility of the nomogram. The X-axis represents the percentage of threshold probability, and the Y-axis represents the net benefit.

Based on independent prognostic factors, we constructed a nomogram by weighing M, sex, stage, grade, T, age and risk score for predicting 1-, 3- and 5-year OS in GC patients (Figure 5A). The results in Figure 5B show that compared with other clinical factors (including risk score), the AUC value of the nomogram was the highest, reaching 0.731, which indicated that the nomogram was the most accurate in evaluating the survival probability of GC patients. Time-dependent ROC curves were used to evaluate the specificity of the nomogram for different survival periods. The 1-, 3-, and 5-year AUCs of the nomogram were 0.731, 0.732, and 0.762, respectively (Figure 5C). The prediction values of the 1-, 3- and 5-year nomograms in the calibration curves were close to the 45-degree line, which indicated that the nomogram demonstrates good prediction capability (Figure 5D). Additionally, the DCA curve further confirmed that the nomogram based on the cuproptosis-related lncRNA risk model had great clinical applicability in estimating the OS of GC patients (Figure 5E).

### Principal component analysis and gene set enrichment analysis

**Figure 6:**
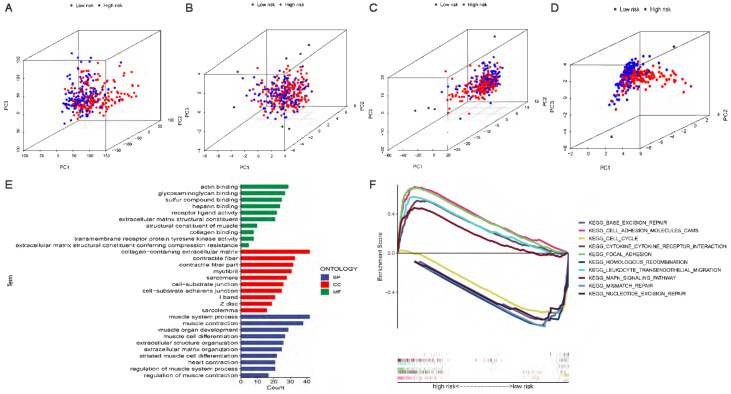
** PCA, GO and KEGG analysis. (A)** PCA of all genes.** (B)** PCA of cuproptosis genes. **(C)** PCA of cuproptosis-related lncRNAs.** (D)** PCA of risk lncRNAs. **(E)** GO enrichment analysis of risk-related genes. **(F)** KEGG enrichment analysis in the high-risk subgroup and the low-risk subgroup.

In four expression profiles (all genes, cuproptosis-related genes, cuproptosis-related lncRNAs, and the 6 cuproptosis-related lncRNAs for constructing the risk model), we performed PCA to examine the differences between the high-risk and low-risk subgroups (Figure 6A-D). The results showed that 6 cuproptosis-related lncRNAs had the best discriminating ability and could distinguish between the high-risk and low-risk subgroups quite well. To investigate differences in biological functions between the high-risk and low-risk subgroups, we performed GO and KEGG analyses using GSEA software in the entire set. GO analysis revealed that cuproptosis-related lncRNAs were associated with cell-cell interactions and ligand-receptor binding (Figure 6E). Figure 6F shows the top 5 pathways that were significantly enriched in the high-risk and low-risk subgroups. The five significantly enriched KEGG pathways in the high-risk subgroup were leukocyte transendothelial migration, cell adhesion molecules CAMs, cytokine-cytokine receptor interaction, MAPK signaling pathway and focal adhesion. The five significantly enriched KEGG pathways in the low-risk subgroup were base excision repair, homologous recombination, mismatch repair, nucleotide excision repair and cell cycle (all p < 0.01; FDR < 0.05; |NES| > 1.5).

### Characteristics of the TME and immune cell infiltration in the high-risk and low-risk subgroups

**Figure 7:**
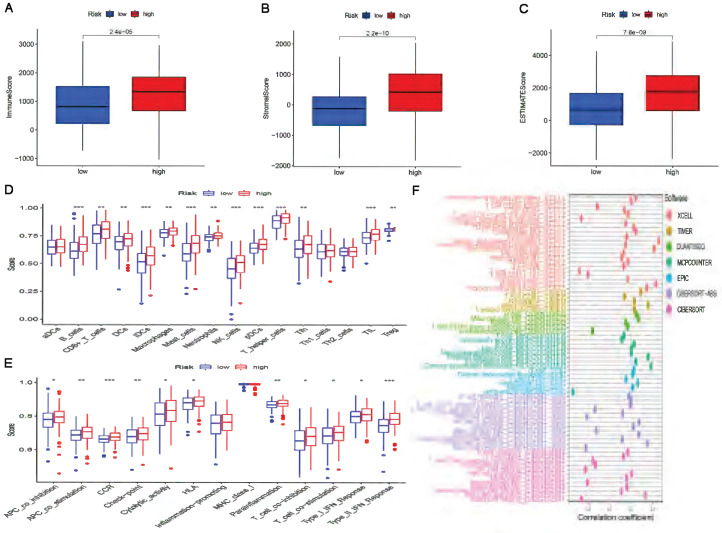
** The landscape of tumor immune infiltration in the high-risk and low-risk subgroups.** Comparison of ImmuneScore **(A)**, StromalScore **(B)**, and ESTIMATEScore **(C)** in the high-risk and low-risk subgroups. Differences in infiltrating immune cell types **(D)** and their immune functions **(E)** between the high-risk and low-risk subgroups based on the ssGSEA scores. **(F)** Correlation analysis between the risk signature and infiltrating immune cells.

Since the results of gene enrichment analysis showed that the high-risk subgroup was associated with multiple immune-related pathways, we further investigated the relationship between risk scores and immune infiltration. First, we calculated TME scores to measure the differences in the extent of infiltrating stromal components and immune cells between the high-risk and low-risk subgroups using the “ESTIMATE” package. As shown in Figure 7A-C, the ImmuneScore, StromalScore, and ESTIMATEScore of the high-risk subgroup were significantly higher than those of the low-risk subgroup. Then, ssGSEA was performed to research the relationship between the risk score and TICs and immune-related function using the “GSEABase”, “GSVA”, “limma”, “reshape2” and “ggpubr” packages. As shown in Figure 7D, B cells, CD8+ T cells, DCs, iDCs, macrophages, mast cells, neutrophils, NK cells, pDCs, T helper cells, Tfh cells, TILs and Tregs were significantly upregulated in the high-risk subgroup. The results in Figure 7F also showed that in the high-risk subgroup, immune-related functions such as APC co-inhibition, APC co-stimulation, CCR, check point, cytolytic activity, HLA, parainflammation, T cell co-inhibition, T cell co-stimulation, Type I IFN response and Type II IFN response were more active than those in the low-risk subgroup. Finally, a bubble chart was created to further verify the association between multiple immune cells and the risk score by using the results predicted by different softwares. The results showed that there was a favorable correlation between the degree of immune cell infiltration and the risk score (Figure 7F).

### The significance of the risk model in TMB and immunotherapy

**Figure 8:**
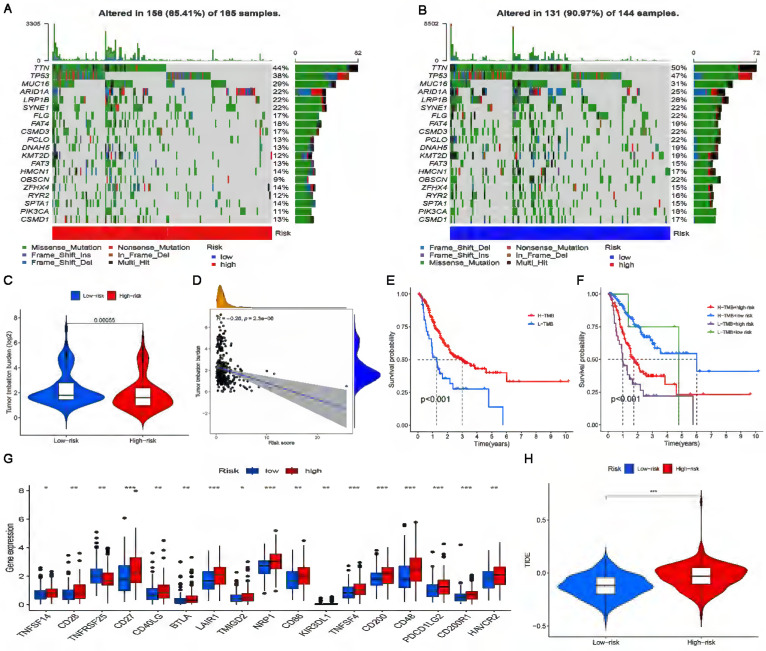
** The significance of the risk model in TMB and immunotherapy. (A, B)** Waterfall plots display mutation information of the genes with high mutation frequencies in the high-risk subgroup and the low-risk subgroup. **(C)** TMB difference in the high-risk and low-risk subgroups. **(D)** Correlation analysis between TMB and risk score.** (E)** Comparison of Kaplan-Meier curves of the high-TMB and low-TMB subgroups. **(F)** Kaplan-Meier curve analysis of OS is shown for patients classified according to the TMB and risk model. **(G)** The difference in the expression of 17 immune checkpoint molecules in the high-risk subgroup and the low-risk subgroup. **(H)** Differences in immunotherapy efficacy between the high-risk and low-risk subgroups.

The landscape of the somatic mutation in GC was explored using the TCGA cohort. We found that although the mutation frequency of each gene in the high-risk subgroup and the low-risk subgroup was different, TTN, TP53 and MUC16 were the top three genes with the highest mutation frequency (Figure 8A, B). The somatic mutation data were used to generate TMB scores to further compare TMB between high-risk and low-risk subgroups.

The results of difference analysis showed that the TMB of the low-risk subgroup was higher than that of the high-risk subgroup (P<0.001) (Figure 8C). In addition, the results of the correlation analysis also showed that the risk score was negatively correlated with TMB (Figure 8D). The GC samples were divided into high-mutation and low-mutation subgroups according to the TMB score. The high-mutation subgroup had a higher OS than the low-mutation subgroup, according to the survival analysis (Figure 8E). According to the risk score and TMB score, GC samples were further divided into four subgroups to compare the survival status of each subgroup. The results showed that the subgroup with low TMB + high risk had the worst prognosis (Figure 8E). Given the differences in TMB in the high-risk and low-risk subgroups, we wanted to further investigate whether there were differences in the response to immunotherapy between the subgroups. To evaluate the response of GC patients to immunotherapy in high-risk and low-risk subgroups, the expression levels of common immune checkpoint molecules were compared. Figure 8G shows that the expression of most immune checkpoint molecules was upregulated in the high-risk subgroup, indicating that GC patients in the high-risk subgroups may benefit more from ICI treatment. TIDE analysis was performed to evaluate the effect of ICI treatment in patients. In general, the lower the TIDE score is, the greater the likelihood of immune escape and the less benefit of immunotherapy. As shown in Figure 8H, patients in the high-risk subgroup had higher TIDE scores than those in the low-risk subgroup.

### Prediction of potential chemical drugs using the risk model

The pRRophetic algorithm was performed to predict the relationship between sensitivity to therapeutic drugs and risk score. The results showed that there were significant differences in sensitivity to 20 conventional chemotherapeutic or targeted drugs used to treat GC between the high-risk and low-risk subgroups (P<0.0001). Among them, the IC50 of 18 conventional chemotherapeutic or targeted drugs was higher in the low-risk subgroup, while the IC50 of only 2 conventional chemotherapeutic or targeted drugs was higher in the high-risk subgroup (Figure S2).

### Consensus clustering of cuproptosis-related lncRNAs and precision medicine in clusters

**Figure 9:**
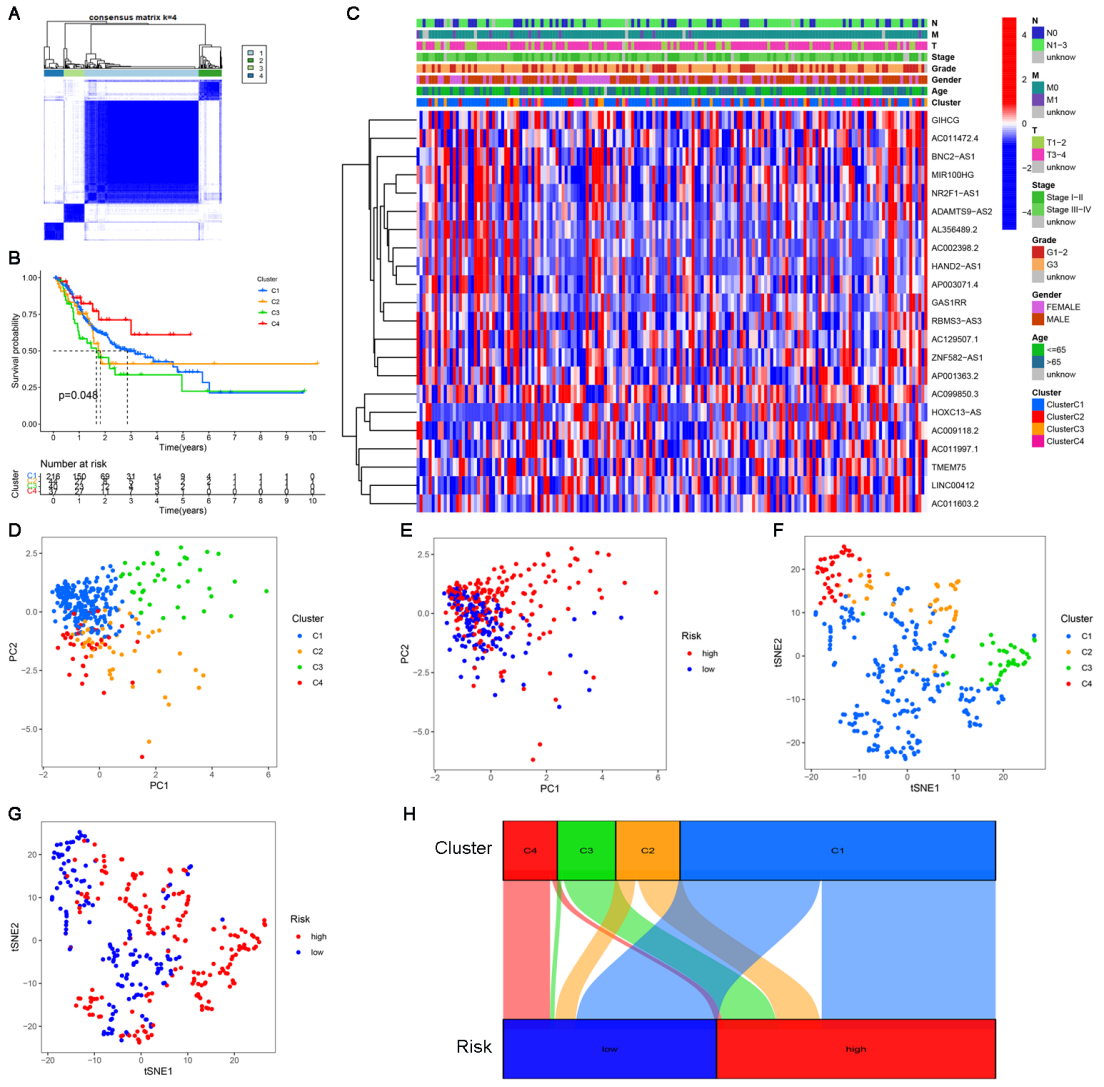
** Consensus clustering analysis of cuproptosis-related lncRNAs. (A)** Consensus clustering matrix for k=4. **(B)** Kaplan-Meier curves of OS for patients with gastric cancer in the four subgroups (P<0.05). **(C)** Heatmap and clinicopathologic features of the four clusters.** (D, E)** The PCA of four clusters and two risk subgroups.** (F, G)** The t-SNE of four clusters and two risk subgroups. **(H)** The Sankey relational diagram of four clusters and two risk subgroups.

**Figure 10:**
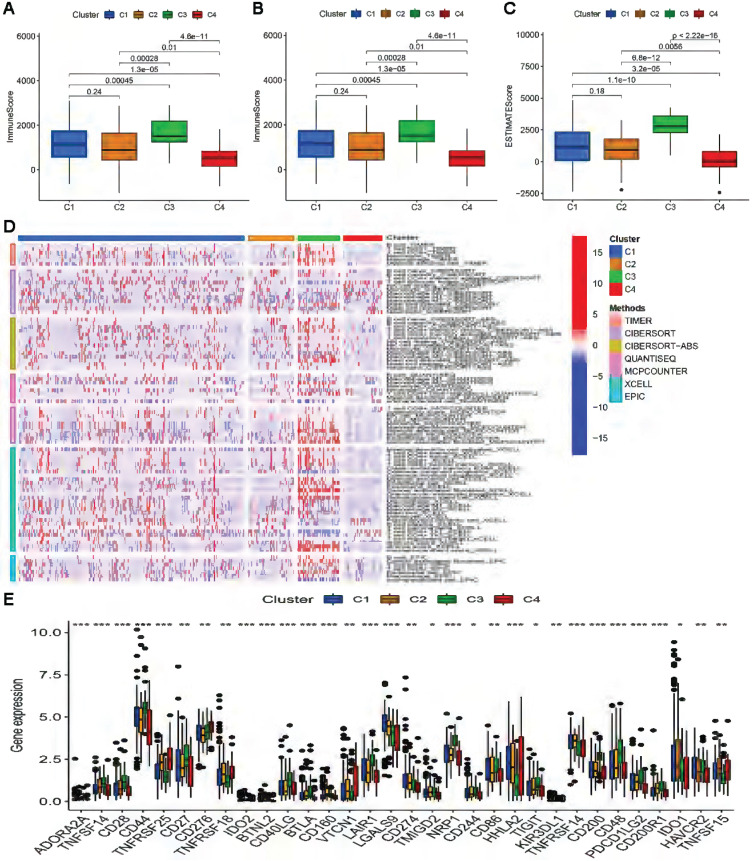
** Difference in immune infiltration levels in clusters and prediction of responses to immunotherapy.** Comparison of ImmuneScore **(A)**, StromalScore **(B)**, and ESTIMATEScore **(C)** in respective clusters. **(D)** The heatmap of immune cells in respective clusters. **(E)** The difference in the expression of 32 checkpoint molecules in respective clusters.

Clusters usually differ in prognosis and their immune microenvironments vary, which is often associated with difference in responses to immunotherapy. Based on the similarity in the expression levels of the 22 cuproptosis-related lncRNAs, we regrouped 337 CC samples into clusters by the ConsensusClusterPlus package. By increasing the clustering variable (k) from 2 to 9, we found that when k = 4, the value of the clustering distribution function (CDF) was the smallest, and the consensus matrix showed a clear boundary (Figure S3, Figure 9A). Kaplan-Meier curve analysis indicated that there were significant differences in survival among the four subgroups, with the lowest OS in Cluster 3 and the highest OS in Cluster 4 (Figure 9B). The clinical features of the four clusters are shown on the heatmap. Compared with the Cluster 4 subgroup, the Cluster 3 subgroup appeared to be associated with a higher grade and stage (Figure 9C). PCA and tSNE were used to distinguish different clusters and high-risk and low-risk subgroups. We found that compared with 2 risk subgroups, 4 cluster subgroups could better distinguish GC samples, which was beneficial to customizing treatment to individual GC patients in the cluster (Figure 9D-G). The Sankey diagram clearly shows the corresponding relationship between each cluster and risk subgroup. As shown in Figure 9H, Cluster 3 corresponded to high-risk subgroups, while Cluster 4 corresponded to low-risk subgroups, which explained why patients in Cluster 3 had the lowest survival and was tended to be in later stages. The difference in tumor immune infiltration among different cluster subgroups was further explored. The results showed that there were significant differences in the ImmuneScore, StromalScore, and ESTIMATEScore among each cluster subgroup, with the highest score in the Cluster 3 subgroup and the lowest in the Cluster 4 subgroup (P<0.001). A heatmap was created to explore the association between tumor-infiltrating immune cells and different cluster subgroups by using the results predicted by different softwares. The results showed that multiple software predictions were comparable, with multiple immune cells most active in the Cluster 3 subgroup (Figure 10D). These findings suggested that the Cluster 3 subgroup had a higher level of immune infiltration than the other cluster subgroups. In addition, we found that most of the immune checkpoint molecules had significant differences in expression levels among cluster subgroups, which may indicate difference in responses to immunotherapy (Figure 10E). Based on these cluster subgroups, we might further study the effects of immunotherapy effects on different patients and improve patient-optimized treatment stragety in GC patients.

## Discussion

In recent years, radiotherapy, chemotherapy, and targeted therapy have made limited progress in the clinic, and the median overall survival for advanced-stage GC is only approximately 8 months 23. As one of the breakthroughs in tumor therapy, immunotherapy has become an effective treatment after surgery, radiotherapy, chemotherapy and targeted therapy. Immunotherapy can be effective even when other treatment has failed or provided limited benefit 24. Reprogramming the TME to an immunostimulatory state, redirecting the cytotoxic effects of cytotoxic lymphocytes toward tumor cells and inducing immunogenic death of tumor cells can transform immune “cold tumors” into “hot tumors”, which is an effective strategy for the treatment of GC 25, 26. However, due to the immunosuppressive state of the TME, some patients have a poor response to immunotherapy 5. Therefore, the correct identification and distinction between immune cold tumors and hot tumors is helpful to strengthen the immunotherapy of tumors. Noninfiltrated tumors with a low TME immune score are usually regarded as cold tumors, while highly infiltrated tumors with a high TME score are regarded as hot tumors. For immune hot tumors, T cell-targeted immunotherapy or other immunotherapeutic drugs can be used to treat patients. However, it is difficult for immune cold tumors because they have low levels of pre-existing infiltrating T cells 27. The effective method is to transform the immune cold tumor into a hot tumor first, rather than giving other treatments directly 28. Recent studies have shown that cuproptosis may be related to the progression of a variety of malignant tumors and the tumor immune microenvironment, which will affect the prognosis and immunotherapy of patients 29-32. However, the relationship between cuproptosis-related lncRNAs expression and tumor microenvironment of GC has not been reported. Therefore, the thorough elucidation of the overall characteristics of the TME regulated by cuproptosis-related lncRNAs is crucial to understand the antitumor immune milieu of the TME and guide the precision treatment of tumors.

In this study, 6 cuproptosis-related lncRNAs (TMEM75, LINC00412, GAS1RR, HOXC13-AS, AC011997.1, and AC129507.1) were used to construct the risk model by LASSO regression. To demonstrate the practical value of the risk model in clinical settings, ROC and C-index curves were used to assess the predictive performance for the OS of GC patients in the entire set. The values of AUC and consistency index indicated that our constructed risk model is more accurate and reliable in predicting GC patient survival than other published risk models 33. Stratified survival analysis, independent prognostic analysis and PCA implied that this model offers high sensitivity for survival prediction. In addition, the ROC, calibration and DCA curves confirmed that the nomogram based on the cuproptosis-related lncRNA risk model had great clinical applicability, which can guide clinicians to judge the prognosis of patients with GC.

Two distinct subgroups were identified in the risk model, which had significant difference in biological functions of the associated genes, TME scores, immune cell infiltration characteristics and immunotherapy responses. We found that the high-risk subgroup was associated with immune activation and tumor-related pathways, while the low-risk subgroup was associated with gene self-repair. Compared with the low-risk subgroup, the high-risk subgroup had higher TME scores. At the same time, the expression of CD4+ T cells, CD8+ T cells, M2 macrophages, mast cells, neutrophils, NK cells, T helper cells, Tregs and dendritic cells in the high-risk subgroup was significantly upregulated, indicating that the high-risk subgroup could be identified as an immune hot tumor. These abnormal infiltrating immune cells in the high-risk subgroup may be correlated with GC development. Through the detection of GC clinical samples, Jin et al. found that high infiltration of CD8 T cells in the tumor predicted poor overall survival and poor response to chemotherapy 34. Studies have shown that M2 macrophages can lead to acceleration of GC cell proliferation and tumor growth in xenograft models 35. It has been reported that the disease-free survival time and total survival time of gastric cancer patients with high immune infiltration are significantly shorter than those with low immune infiltration 36. Further analysis showed that the molecular activity of common immune checkpoint molecules was higher in the high-risk subgroups and that the response of patients to ICI treatment was better, which was consistent with the characteristics of immune hot tumors. In addition, the sensitivity of 20 common conventional chemotherapeutic or targeted drugs in the high-risk and low-risk subgroups was compared to guide clinicians in selecting appropriate anticancer drugs for GC patients.

With in-depth research on tumor immunology and precision medicine, precision immunotherapy has become a new direction of tumor therapy 37, 38. The key to this precise immunotherapy is to identify the right patients and provide specific treatments, including ICIs, therapeutic antibodies, and cell therapy. The classification of immune cold tumors and hot tumors is a feasible way to identify patients who might benefit from immunotherapy 26, 27. It has been reported that clusters usually differ in prognosis and their immune microenvironments vary, which is often associated with difference in responses to immunotherapy 39. To accurately identify immune cold tumors and hot tumors, all samples of GC were divided into 4 distinct clusters by consensus clustering. The Sankey diagram showed that Cluster 3 belongs to the high-risk subgroup (immune-inflamed phenotype), and Cluster 4 belongs to the low-risk subgroup (immune-desert phenotype). The immune-inflamed phenotype, also known as an immune hot tumor, is characterized by abundant immune cell infiltration and higher TME scores, which was consistent with our results 40. Therefore, patients with the immune-inflamed phenotype (Cluster 3) might benefit from immunotherapy or combined immunotherapy. The immune-desert phenotype, also known as a cold tumor, is characterized by immunosuppression, lack of activated T cells and other related immune cells, and rapid tumor growth 40, 41. Rapid tumor growth can affect the growth and infiltration of immune cells in the TME, which is not conducive to T cell proliferation and activation, resulting in poor prognosis and poor response to immunotherapy 40, 41. The results of the TME score, characteristics of immune cell infiltration and response to ICI treatment confirmed the reliability of our immunophenotypic classification of the two distinct clusters, which further strengthened our logic behind precision treatment of gastric cancer according to different types of patients.

The current study has several limitations, although multiple methods were used to evaluate the model. First, although we selected a catalog of 16 known cuproptosis-related genes, new cuproptosis-related genes need to be enrolled to optimize the accuracy of the risk model. Our study, as a retrospective study, may be susceptible to the inherent biases of this research paradigm 42. Therefore, we need to collect more clinical data or conduct research on prospective cohort of GC patients undergoing immunotherapy to validate our findings. Finally, our data are from TCGA, and all analyses are based on this, which may also lead to bias. If we perform a comprehensive analysis of data from other sources and reproduce our findings, our results will be more reliable.

## Conclusion

In this study, we systematically evaluated the value of cuproptosis-related lncRNAs in predicting survival, estimating the role of the tumor microenvironment and immune cell infiltration, deducing potential compounds for the treatment of CC and precision immunotherapy in the clusters. The signature of six lncRNA associated with cuproptosis could predict the survival of GC patients, and the identification of immune cold tumors and hot tumors will contribute to the individualized and precise treatment of cancer patients in the future.

## Supplementary Material

Supplementary figures.

## Abbreviations

GC: gastric cancer
STAD: stomach adenocarcinoma
TME: tumor microenvironment
lncRNA: long non-coding RNA
TCGA: The Cancer Genome Atlas
LASSO: least absolute shrinkage selection operator
ROC: receiver operating characteristic
PCA: principal component analysis
DCA: decision curve analysis
AUC: area under curve
ICIs: immune checkpoint inhibitors
PCD: programmed cell death
GTF: gene transfer format
FPKM: fragments per kilobase million
OS: overall survival
GSEA: gene set enrichment analysis
TICs: tumor-infiltrating immune cells
ssGSEA: single sample gene set enrichment analysis
TMB: tumor mutational burden
TIDE: Tumor Immune Dysfunction and Exclusion
IC50: the half-maximal inhibitory concentration
GDSC: Genomics of Drug Sensitivity in Cancer
t-SNE: t-distributed stochastic neighbor embedding
