# Supplementary material for: Distinct tumor microenvironment landscapes in gastric cancer classified by cuproptosis-related lncRNAs
Source: J Cancer. 2022 Dec 7;13(15):3687–700. doi: 10.7150/jca.79640 (PMC9809317; doi:10.7150/jca.79640)
Supplement: Supplementary file 1 — Supplementary figures. [file jcav13p3687s1.pdf]

Supplementary Figure

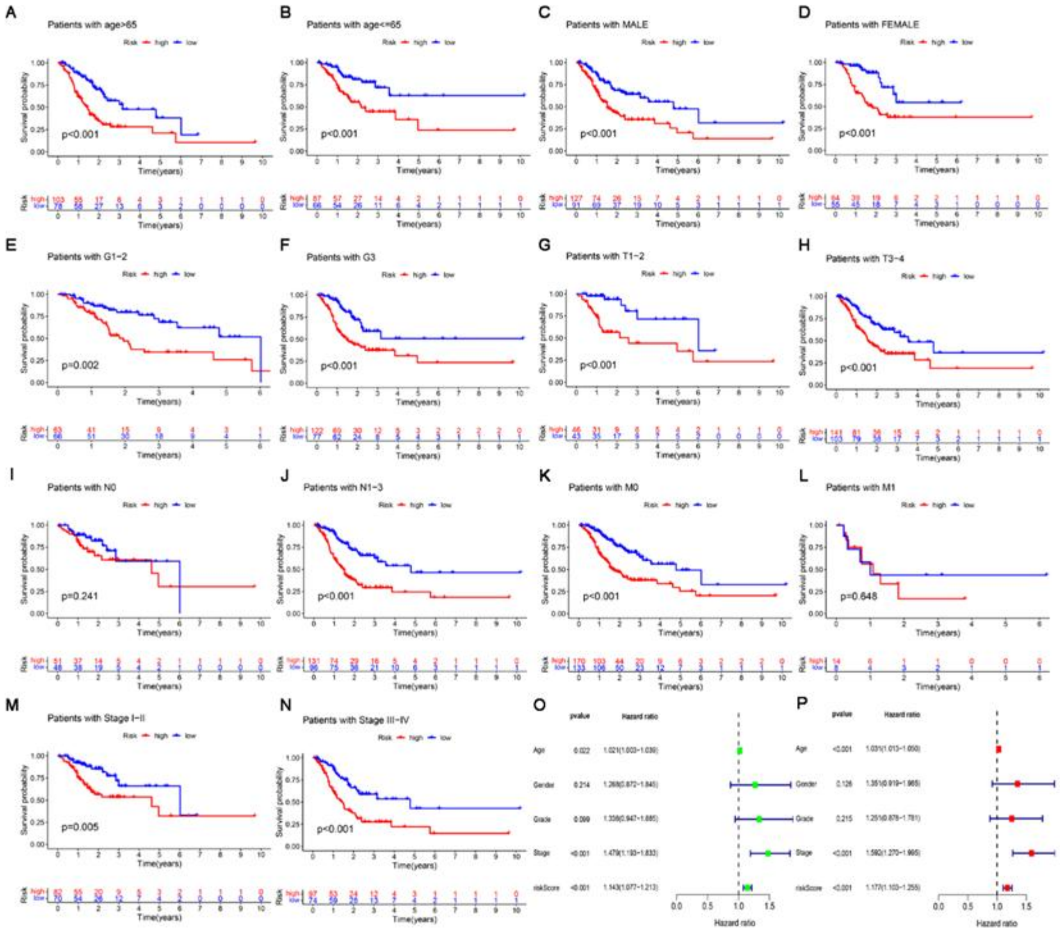

Figure S1. Prognostic value of the cuproptosis-related lncRNA risk model in the entire set. (A-N) Kaplan–Meier survival curves for the high- and low-risk subgroups stratified by clinical characteristics. Univariate (O) and multivariate (P) independent prognostic analyses in the entire set.

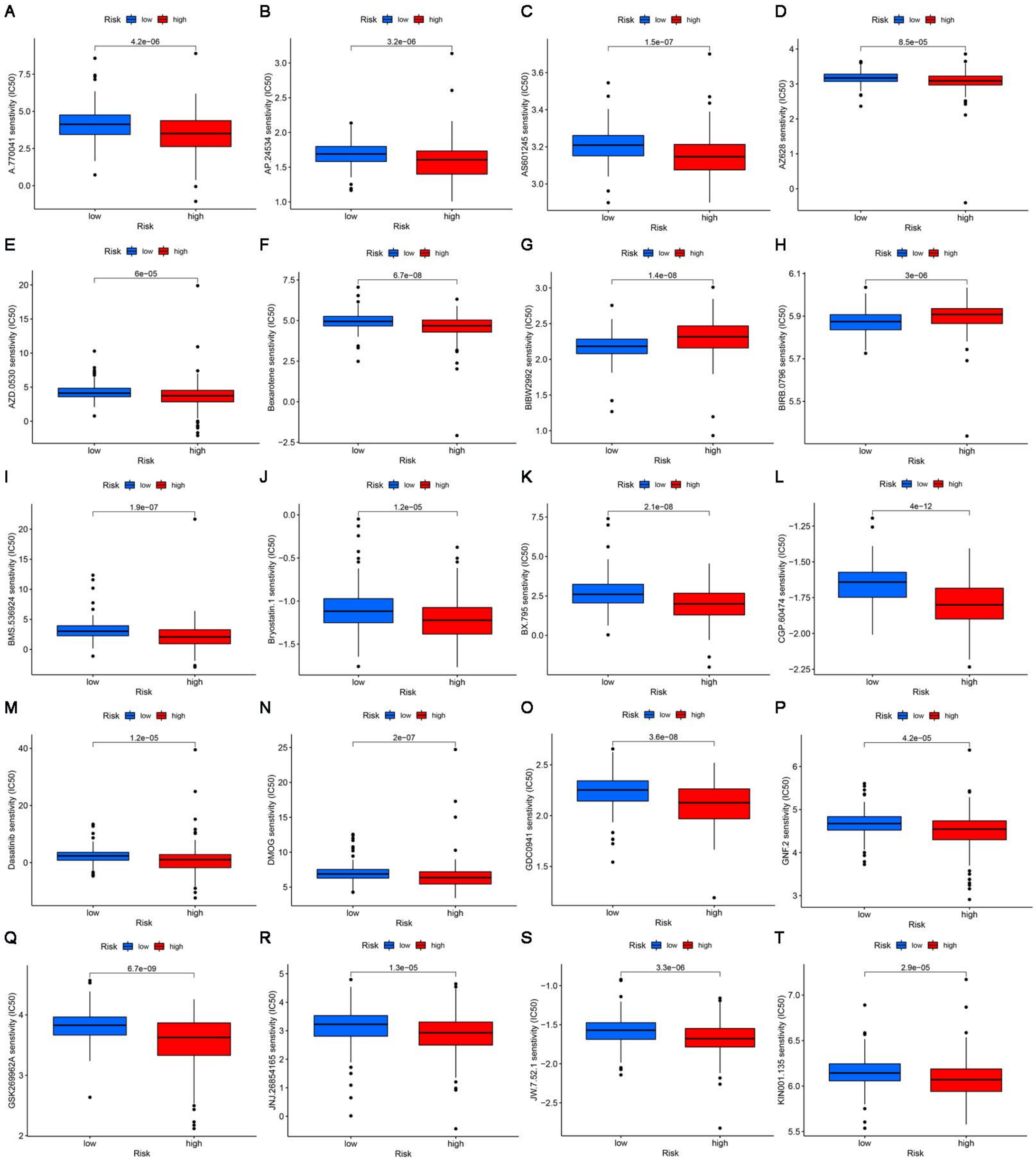

Figure S2. Relationships between risk score and chemotherapeutic sensitivity.

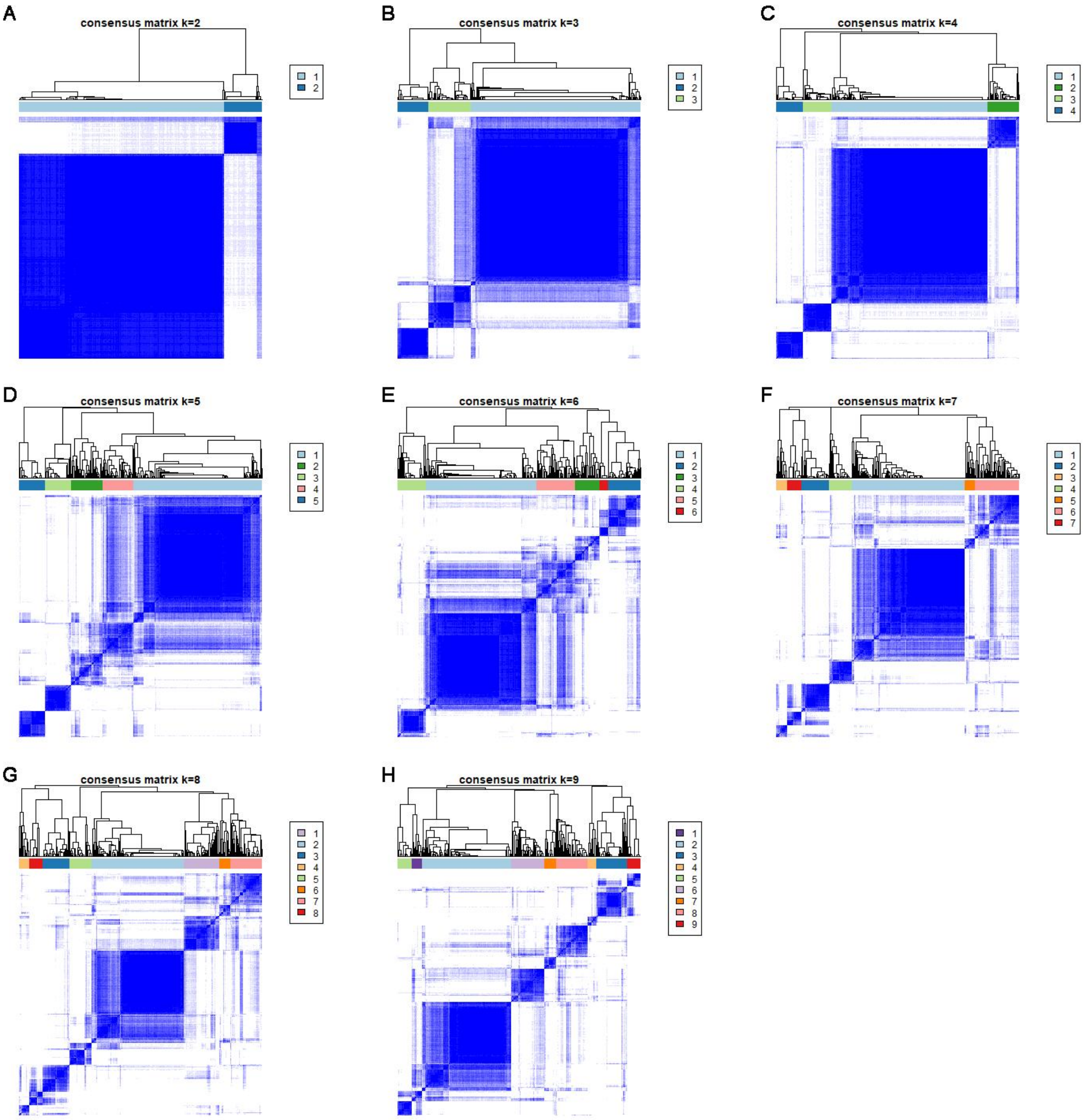

Figure S3. The clustering variable (k) from 2 to 9.
